# Ultrasensitive detection of cadmium ions using a microcantilever-based piezoresistive sensor for groundwater

**DOI:** 10.3762/bjnano.11.108

**Published:** 2020-08-18

**Authors:** Dinesh Rotake, Anand Darji, Nitin Kale

**Affiliations:** 1 Department of Electronics Engineering, Sardar Vallabhbhai National Institute of Technology, Surat, Gujarat, India; 2 The Chief Technology Officer, NanoSniff Technologies Pvt. Ltd., F-14, 1st Floor, IITB Research Park, Old CSE Building, IIT Bombay, Powai, Mumbai – 76, India.

**Keywords:** BioMEMS, heavy metal ions (HMIs), limit of detection (LOD), microcantilevers, microfluidics, micro-electromechanical systems (MEMS), piezoresistive sensors, SAM (self-assembled monolayers), World Health Organization (WHO)

## Abstract

This paper proposes the selective and ultrasensitive detection of Cd(II) ions using a cysteamine-functionalized microcantilever-based sensor with cross-linked ᴅʟ-glyceraldehyde (DL-GC). The detection time for various laboratory-based techniques is generally 12–24 hours. The experiments were performed to create self-assembled monolayers (SAMs) of cysteamine cross-linked with ᴅʟ-glyceraldehyde on the microcantilever surface to selectively capture the targeted Cd(II). The proposed portable microfluidic platform is able to achieve the detection in 20–23 min with a limit of detection (LOD) of 0.56 ng (2.78 pM), which perfectly describes its excellent performance over other reported techniques. Many researchers used nanoparticle-based sensors for the detection of heavy metal ions, but daily increasing usage and commercialization of nanoparticles are rapidly expanding their deleterious effect on human health and the environment. The proposed technique uses a blend of thin-film and microcantilever (micro-electromechanical systems) technology, which mitigate the disadvantages of the nanoparticle approaches, for the selective detection of Cd(II) with a LOD below the WHO limit of 3 μg/L.

## Introduction

Water is fundamentally essential for sustaining life, and an increase in the global population has led to an exponential increase in waste disposal, which causes significantly increased requirements regarding the control of water quality [1]. Clean water is one of the main priorities of the 21st century worldwide, and negligence to this may have a significant effect on maintaining the safety and security of human beings [2–3]. One common water contamination is caused by cadmium ions. There are numerous sources of Cd ions in groundwater, including industrial wastewater, mining industry, fossil fuels, iron and steel industry, cement manufacturing units, electroplating industry, manufacturing units of PVC, Ni–Cd batteries, fertilizers, pesticides, photovoltaic devices, soil, and sediments. Cadmium is a highly toxic heavy metal ion (HMI). Cadmium poisoning may cause fatigue, headaches, nausea, vomiting, abdominal cramps, bone degeneration, diarrhea, osteoporosis, renal dysfunction, cancer, anemia, and neurological disorders such as Parkinson's disease or Alzheimer's disease [4–5]. The WHO has set a water contamination limit of 3 μg/L Cd(II) [6]. We conclude from the WHO limit that cadmium is hazardous, and smaller Cd concentrations below the limit is also hazardous. Hence, it is essential to sense Cd(II) in the picomolar (pM) range well below the specified WHO limit.

The ion-selective electrodes (ISEs) fabricated by [7] are stable and precise for HMI detection, but the measurement requires by laboratory equipment. Sensors based on nanotubes, nanorods, nanoneedles, or nanoplates are also used to detect HMIs selectively down to the nanomolar range [8–11]. Many authors used adsorption methods to extract heavy metal ions from groundwater [12–16]. However, this is only useful when a pollution source has been already identified. Sensors based on luminescence or fluorescence sensors have been used by many researchers to selectively detect HMIs [17–22]. However, this method also requires laboratory equipment for analysis and detection. Also, most of the reported fluorescent probes reply only on absorption and fluorescence change and need dynamic acquisition [23]. A magnetic field powered pressure sensor proposed by Khan et al. [24] is capable of measuring pressure in the range of kilopascals but the suitability for the very low pressure caused by HMIs needs to be examined. A reduced graphene oxide (RGO)-based sensor and a microfluidic platform fabricated by [25–27] can be used with some surface modification for HMIs, but it is mostly capable of detecting in the micromolar range. A polymer-based microcantilever using an encapsulated piezoresistor has been proposed by Kale et al. [28], but it is not suitable for other high-temperature sputtering processes. Microcantilevers based on SiO_2_ have been manufactured by Tang et al. [29] to enhance the sensitivity of cantilever sensors. Many authors use optical setups for microcantilevers. However, an optical output has several disadvantages during operation in water when the refractive index of water changes [30–31].

Many authors have proposed electrode-based approaches for the selective sensing of Cd(II) [32], but the limits of detection were always in the micromolar to nanomolar ranges. Some of the authors used fluorescent [5,33] and calorimetric [34] approaches to selectively detect the Cd(II). But these approaches required laboratory equipment for analysis and the LODs were also in the nanomolar range. All these methods are reliable for the qualitative and quantitative determination of Cd(II), but they are time-consuming, expensive, and not suitable for on-site determination. The calorimetric approach proposed by [34–37] is free from these problems but not capable of differentiating between two nearby ranges and the LOD is also on the higher side. The electrochemical sensor described in [38] is a good approach, but it also requires a lab instrument for measurement.

We have previously investigated SAMs of homocysteine (HCys) and pyridinedicarboxylic acid (PDCA) for the selective sensing of Hg(II) ions using a portable piezoresistive platform [39–40]. Experimental results confirmed that proposed setup is capable of sensing in the picomolar range. In this paper, we have used the previously designed portable piezoresistive platform for the selective capture Cd(II) in the picomolar range. Preliminary results show that the fabricated device has an excellent response within 20–23 minutes with 0.56 ng/mL (2.78 pM) LOD, which is well below the WHO limit for cadmium ions.

The paper describes the methodology, the formation of SAMs and their characterization using field-emission scanning electron microscopy (FESEM), the use of the portable experimental platform with the MEMS-based piezoresistive device to selective capture Cd(II) at the picomolar level and the verification of the experimental results using energy-dispersive X-ray spectroscopy (EDX).

## Fabrication and Calibration of the Piezoresistive Device

**Figure 1:**
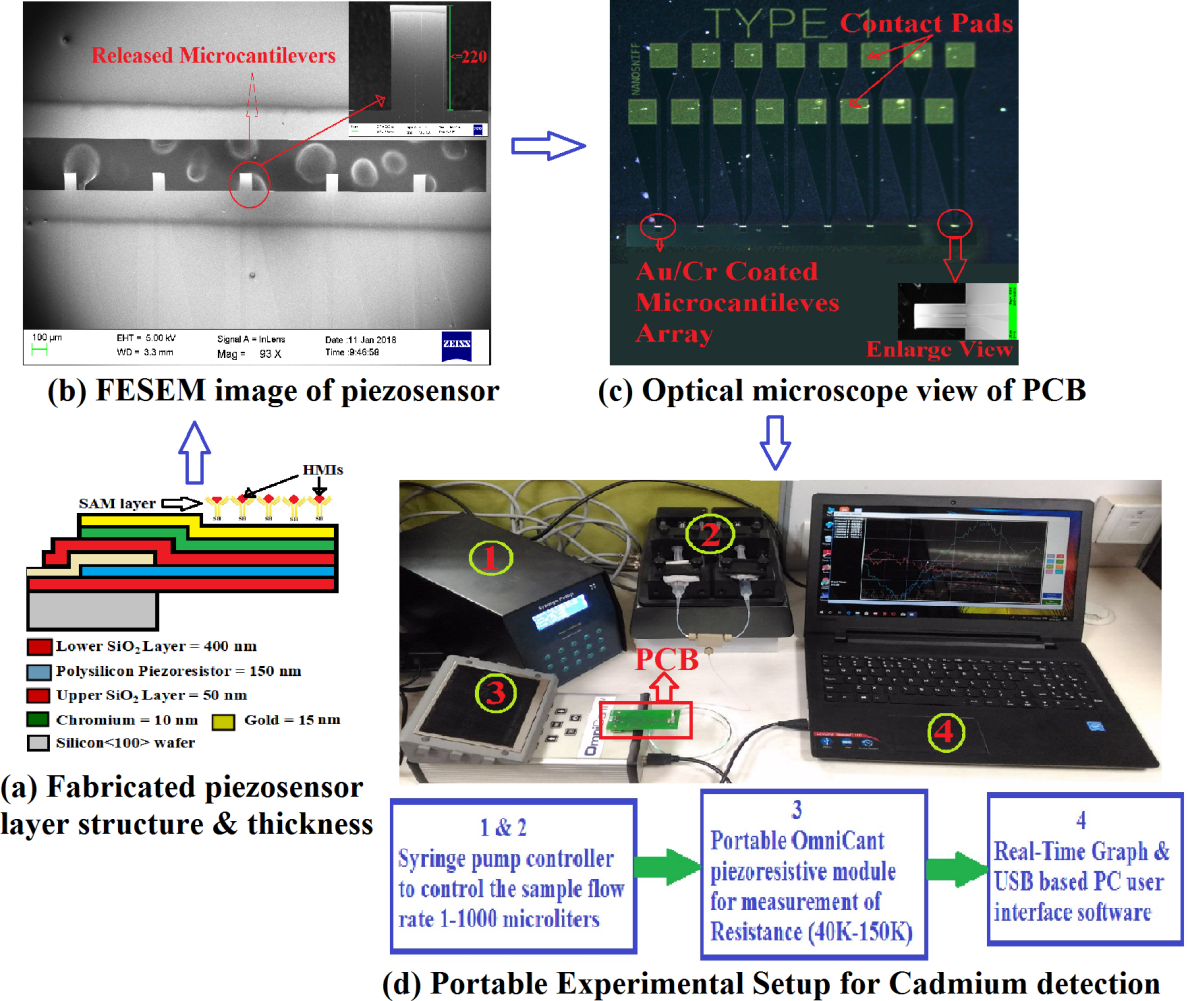
Fabricated piezoresistive sensor and experimental platform for Cd(II) detection.

Previously, a polysilicon-based piezoresistive sensor was fabricated using a standard microfabrication process. It was calibrated using atomic force microscopy (AFM) [40]. The process begins with thermal oxidation of Si at 1000 °C using an oxidation furnace to obtain a thermally grown SiO_2_ layer followed by masking and etching to get the desired pattern. The polysilicon is deposited in a low-pressure chemical vapor deposition (LPCVD) furnace at 630 °C and boron doping (10^18^ per cm^3^) is carried out using ion implantation at 35 keV. The upper SiO_2_ layer is formed by re-oxidizing the polysilicon in an oxidation furnace [40]. The stiffness (*k*) of the fabricated piezoresistive sensor measured using AFM is 131–146 mN/m, which is well below the stiffness required for BioMEMS applications (1000 mN/m [41–42]). COMSOL 5.3 software is used to perform design and simulation of the piezoresistive sensor to optimize the dimensions for better stiffness and sensitivity [43]. The fabricated piezoresistive sensor layer structure with thickness, FESEM image, PCB, and the experimental platform is shown in Figure 1.

For using the microcantilever device for selectively detecting Cd(II) a surface modification is required. The surface modification of the sensor is basically a selective thiol coating on top of a gold surface. Here, SAMs of cysteamine–glyceraldehyde were created on top of microcantilever-based sensors with integrated piezoresistive readout to get the change in resistance due to changes in surface stress. Until now, many people have used lab-based optical setups to measure the change in surface stress of the cantilever sensors. Moreover, the proposed piezoresistive device has capabilities to directly capture the surface stress make this a better option for HMI applications.

## Microfluidic Platform with Piezosensor

**Figure 2:**
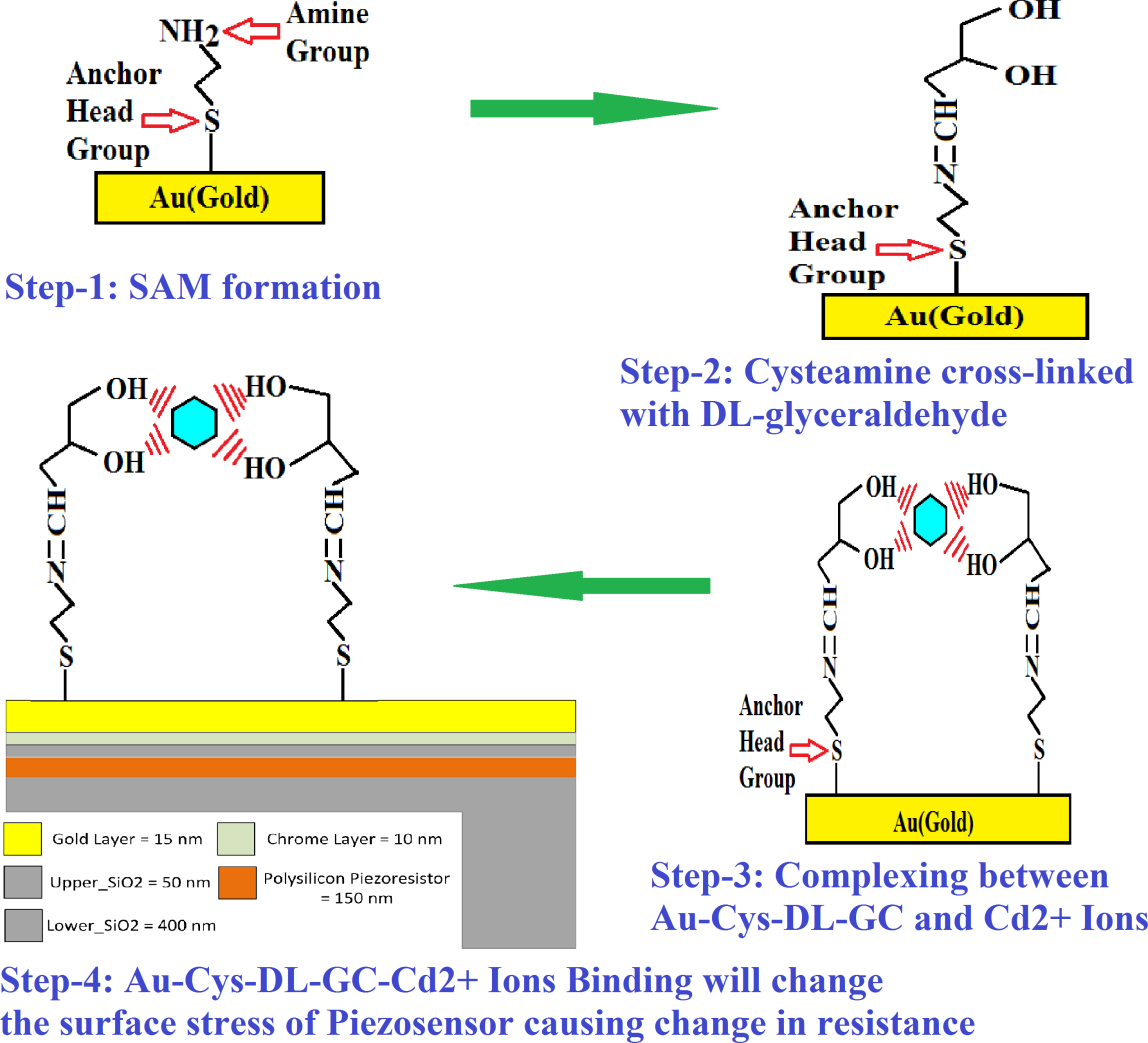
Process flow of biosensor for selective detection of Cd(II) ions.

In the proposed method, the benefits of three different technologies are combined, namely thin film, nanoparticles (NPs), and MEMS, to selectively target Cd(II) in the picomolar range. Also, excessive commercialization of nanoparticles leads to increasing their harmful effect on life and the environment by [44–46]. In this article, an attempt is made to expand the AuNP-based technology proposed by [34] for the ultrasensitive sensing of Cd(II) with cysteamine-functionalized ᴅʟ-glyceraldehyde (Cys-DL-GC) using the advanced MEMS-based piezoresistive platform. The MEMS-based sensor has very high sensitivity compared to any other technique. The complete process flow and the sensing scheme for the piezoresistive microcantilever-based biosensor is shown in Figure 2.

Here, the fabricated microfluidic platform with a microcantilever-based piezoresistive sensor is used to capture Cd(II) in the picomolar range. The amine group (–NH_2_) of cysteamine has an affinity to all types of HMIs and it needs to be cross-linked for selectivity. We have cross-linked the amine group with ᴅʟ-glyceraldehyde at pH 7 to obtain a free –OH group (a Lewis acid) for capturing Cd(II). The pKa value of 12.6 of ᴅʟ-glyceraldehyde yield a strong copmplex of the –OH group with cadmium [34] after blocking the –NH_2_ group of cysteamine.

## Results and Discussion

**Figure 3:**
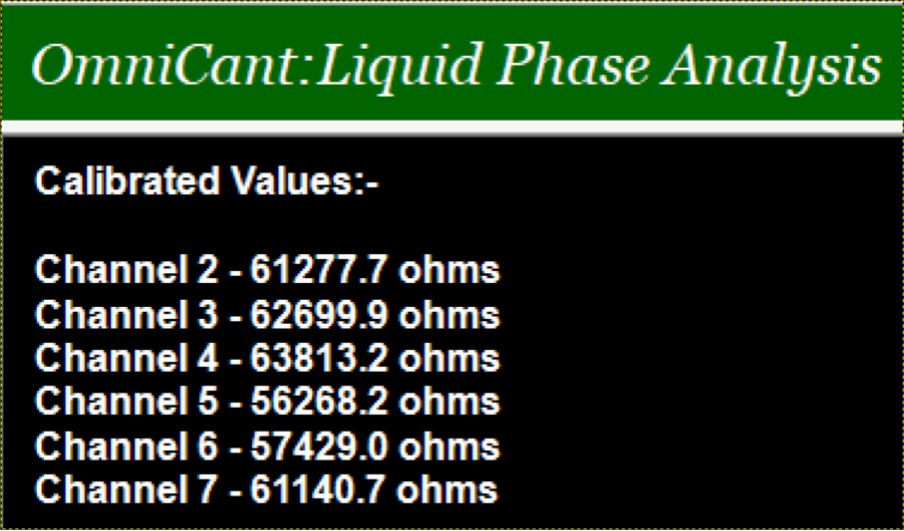
Non-stress calibrated values for used piezoresistive die.

The performance of the fabricated device in the selective detection of Cd(II) in a microfluidic environment is evaluated using the OmniCant setup shown in Figure 1d. The non-stress calibrated resistance values of the piezoresistive sensor using SAMs of cysteamine cross-linked with ᴅʟ-glyceraldehyde (Cys-DL-GC) is in the range of 56268–63813 Ω. The non-stress resistance values of the fabricated piezoresistive die in the OmniCant microfluidic platform are shown in Figure 3. The microcantilever in channel 2 was blocked with acetyl chloride and selected as a reference for the Cys-DL-GC experiments. The values shows that the microcantilevers in channels 1 and 8 broke during the wire bonding and are not present in the analysis. The piezoresistive MEMS devices exhibit a fast response to changes in the resistance depending on the additional mass of Cd(II) loaded on the surface.

### Results of the SAM detecting cadmium ions

The proposed microfluidic platform provides real-time monitoring of Cd(II) in groundwater. We performed the experiment using the coating of cysteamine thiol with cross-linked ᴅʟ-glyceraldehyde (Cys-DL-GC). We have already discussed that the methods based on Au/Ag nanoparticles require the laboratory equipment such as fluorescence spectroscopy, which ultimately leads to a non-portable platform. Hence, our primary focus is the selective detection of the Cd(II) using the fabricated portable experimental platform.

We used the following experimental procedure. A stock solution of cysteamine ([Cys] = 10 mM/10 mL) was prepared. The piezoresistive devices were gently dipped into a petri-dish containing cysteamine thiol for at least 12–24 hours. Longer times yield a better packing density of the SAM. In addition, a 2% (0.2 g/10 mL) solution of ᴅʟ-glyceraldehyde (DL-GC) in phosphate buffer saline (PBS, pH 7) according to [34] was prepared. The cysteamine SAM was allowed to cross-link with the ᴅʟ-glyceraldehyde solution for at least 2–3 hours by covering the container using silver foil. Stock solutions of 1 mM/10 mL of different salts (AlCl_3_, MnCl_2_, CrCl_3_, HgCl_2_, PbCl_2_, CdCl_2_) were used for the experiments. The flow rate was kept constant at 30 μL/min during the experiments. Before the measurement, DI water was used to stabilize the microcantilever in a liquid environment for a period of 7 min. Subsequently, the different heavy metal ion solutions were injected separately and the corresponding change in piezoresistance was measured. The Cd(II) solution was injected and the corresponding change in piezoresistance was measured.

**Figure 4:**
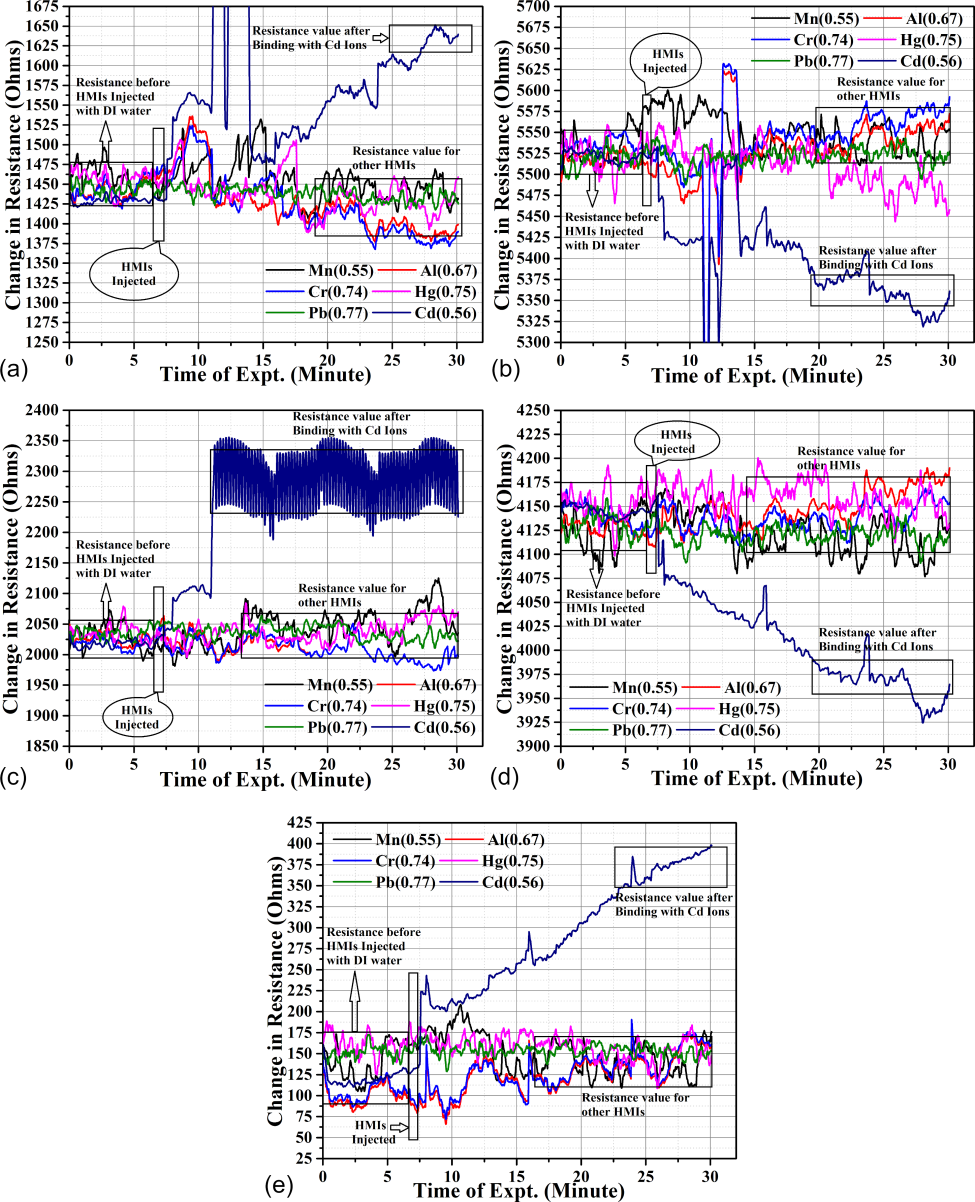
The change in piezoresistance of the unblocked cantilevers with respect to the cantilever blocked with acetyl chloride(channel 2): (a) Cys-DL-GC (channel 3), (b) Cys-DL-GC (channel 4), (c) Cys-DL-GC (channel 5), (d) Cys-DL-GC (channel 6) and (e) Cys-DL-GC (channel 7).

The change in piezoresistance (Δ*R*) is calculated using a formula:

[1] $$\Delta R=\left[\Delta R_{\text{Block}}-\Delta R_{\text{Unblock}}\right],$$

where Δ*R*_Block_ is the change in piezoresistance of the microcantilever blocked with acetyl chloride and Δ*R*_Unblock_ is the change in piezoresistance of the unblocked microcantilever. The change in piezoresistance of unblocked microcantilever compared to that of the blocked microcantilever of acetyl chloride (channel 2) is shown in Figure 4.

Initially, we used DI water to stabilize the microcantilever for a period of 7 min. The change of piezoresistance remained constant during this period. When the different heavy metal ion solutions (except Cd(II)) were injected after 7 min, the change in piezoresistance was minimal (5–30 Ω). When cadmium ions were injected after 7 min, the change in piezoresistance was around 200–300 Ω for each microcantilever. These results show the selectivity of the proposed method for Cd(II) with respect to other HMIs. It is also evident that the microcantilever in channel 5 (Figure 4c) shows a non-linear response. The rationale behind this is that no binding sites are available for cadmium ions on the microcantilever surface. Two microcantilevers exhibit a decrease in resistance because of tensile stress due to a small number of biomolecules (Cd(II)) binding to the surface (Figure 4b,d), while the other three microcantilevers exhibit an increase in the resistance because of compressive surface stress when a large number of biomolecules bind to the microcantilever surface (Figure 4a,c,e) [47].

**Figure 5:**
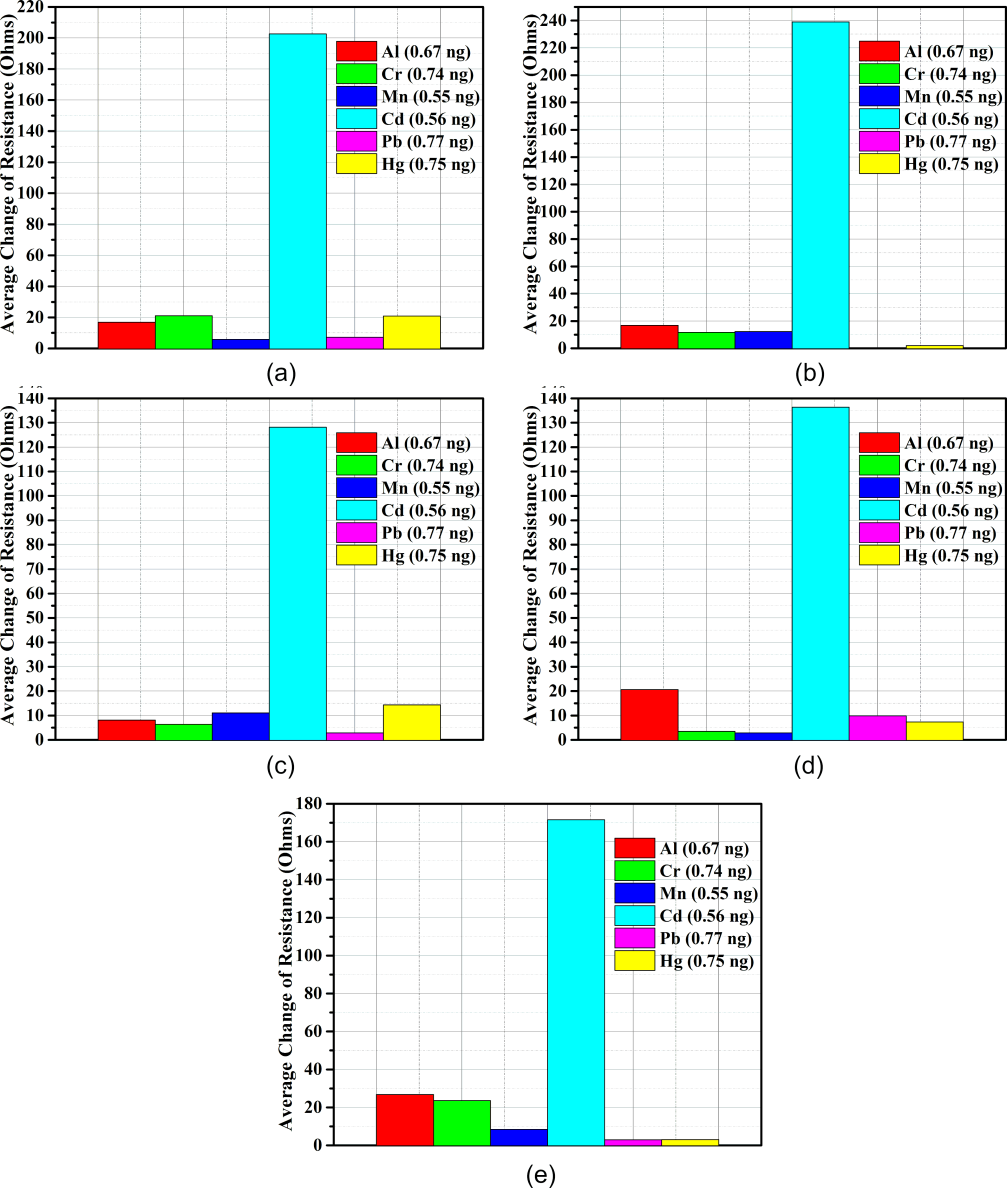
The average change in piezoresistance of microcantilevers: a) Au-Cys-DL-GC(3), b) Au-Cys-DL-GC(4), c) Au-Cys-DL-GC(5), d) Au-Cys-DL-GC(6) and e) Au-Cys-DL-GC(7).

Figure 5 demonstrates the average change in piezoresistance of a sensor based on Au-Cys-DL-GC-coated cantilevers for different heavy metals (AlCl_3_, MnCl_2_, CrCl_3_, HgCl_2_, PbCl_2_, CdCl_2_).

The average value of change in piezoresistance is the difference between the average change in piezoresistance for DI water and the particular heavy metal injected. Our results show that the SAM of cysteamine with cross-linked ᴅʟ-glyceraldehyde(Cys-DL-GC) has a higher selectivity for Cd(II) than for other heavy metals. The average value of change in piezoresistance of the Au-Cys-DL-GC-coated microcantilevers is approximately 130–240 Ω for Cd(II) and 5–30 Ω for the other injected heavy metals. The total value of the average change in piezoresistance for a concentration of 0.56 ng Cd(II) is 877.72 Ω.

### Characterization using Fourier-transform infrared spectroscopy (FTIR)

**Figure 6:**
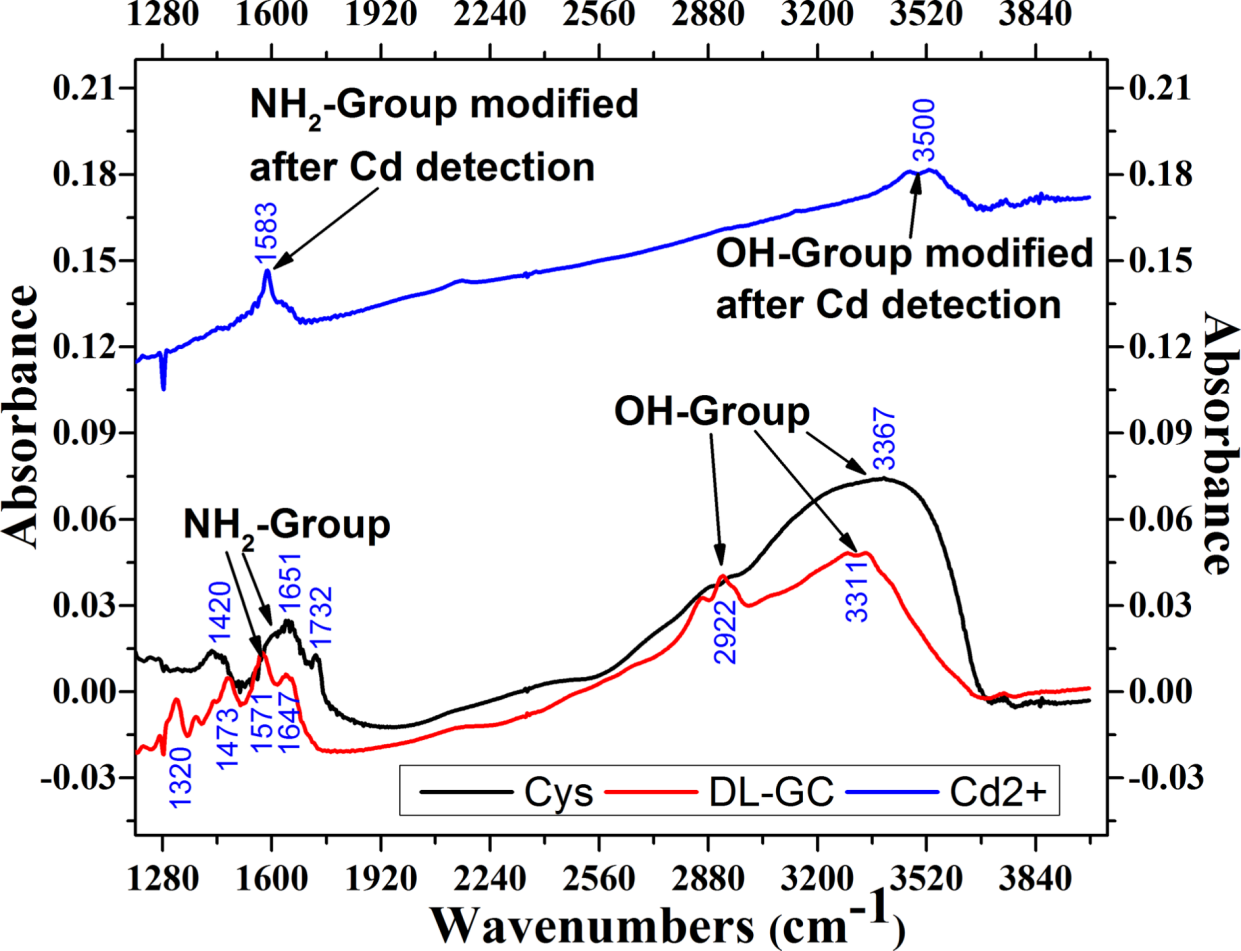
FTIR absorbance spectra of a Cd(II)/DL-GC/Cys/Au/Ti coating.

FTIR is a mature technique for elemental analysis and the identification of functional groups. The FTIR results show –OH stretching in the range of 2900–3750 cm^−1^ and N–H bending (1350–1750 cm^−1^) [48–51]. The FTIR analysis of a Cd(II)/DL-GC/Cys/Au/Cr coating is shown in Figure 6.

The FTIR results of the Cys/Au/Cr coating show a single band at 3367 cm^−1^ associated to the –OH group and three bands at 1420, 1651, and 1732 cm^−1^ associated to the –NH_2_ group. After coating with ᴅʟ-glyceraldehyde (DL-GC/Cys/Au/Cr), the FTIR spectra show four bands at 1320, 1473, 1571, and 1647 cm^−1^ associated to the –NH_2_ group and two bands at 2922 and 3311 cm^−1^ associated to the –OH group. After exposure to Cd(II) two bands related to –OH disappear and only single band at 3500 cm^−1^ is present due Cd(II) binding to the –OH groups. Similarly, for –NH_2_ group), three bands disappear, and only single modified band is present at 1583 cm^−1^ due Cd(II) binding. This modification of the FTIR spectra after exposure to Cd(II) indicates the selective binding of cysteamine cross-linked ᴅʟ-glyceraldehyde (Cys-DL-GC) to cadmium.

### Verification of performed experiment results using EDX

**Figure 7:**
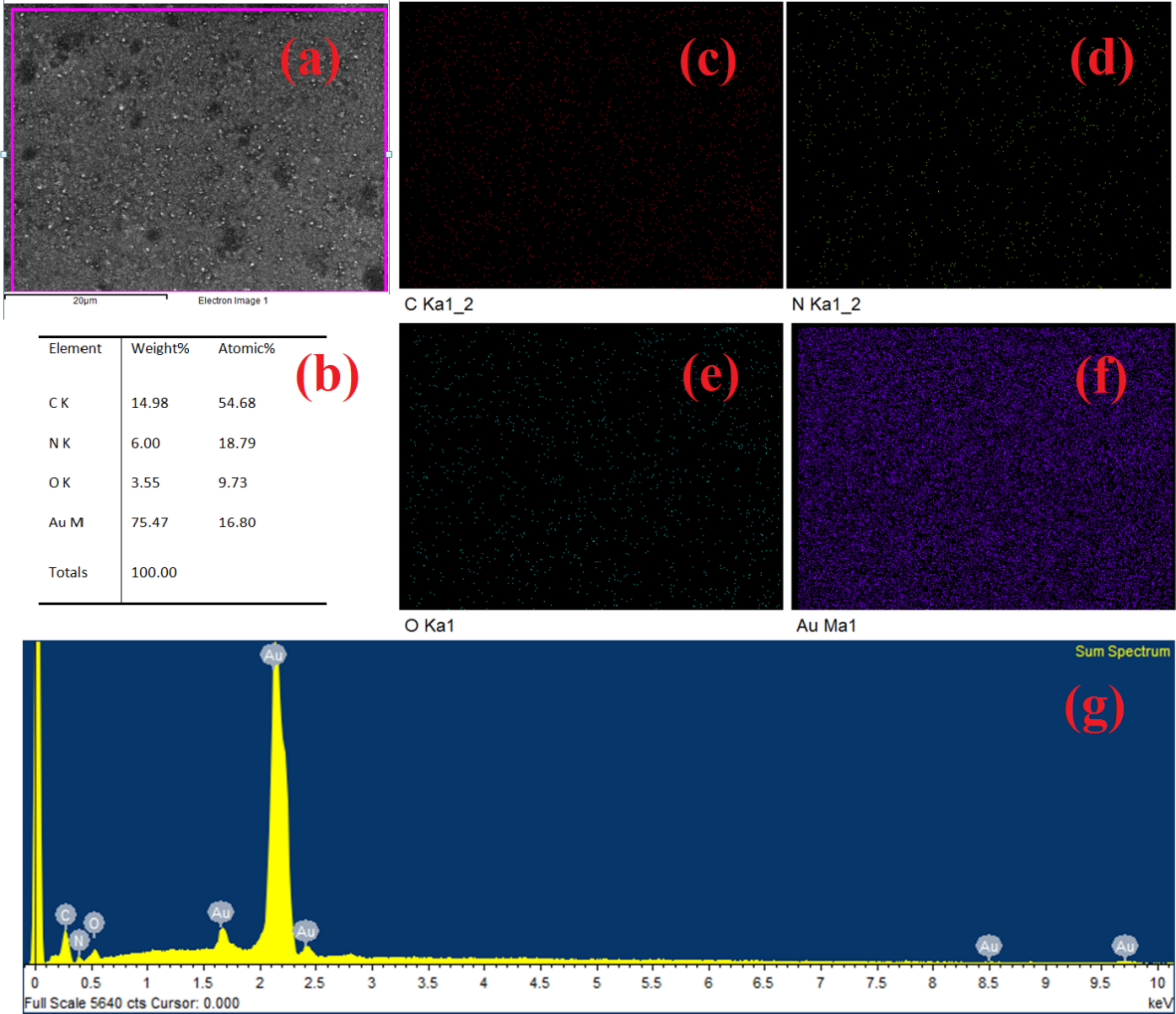
EDX measurement of a SAM of cysteamine (Cys)-cross-linked ᴅʟ-glyceraldehyde (Cys-DL-GC). (a) Scan area for analysis, (b) table with detected elements, (c–f) color mappings of detected elements, (g) EDX spectrum.

In general, the thiol groups can bind to all types of HMIs. Thus, to selectively bind and detect Cd(II) the thiols goups need to be modified or functionalized with materials that are selective for Cd(II). The experimental results show that the fabricated MEMS-based sensor is capable of selective Cd(II) detection using SAMs of cysteamine with cross-linked ᴅʟ-glyceraldehyde (Cys-DL-GC). To characterize the SAM on the microcantilever device only a few analytical techniques are available because of the fragile nature of the cantilever. FESEM/EDX is the preeminent tool to characterize the SAM on the top of the cantilever without damaging the device. The EDX measurement of the sensor with a SAM of cysteamine cross-linked ᴅʟ-glyceraldehyde (Cys-DL-GC) on top of a Au surface is shown in Figure 7.

**Figure 8:**
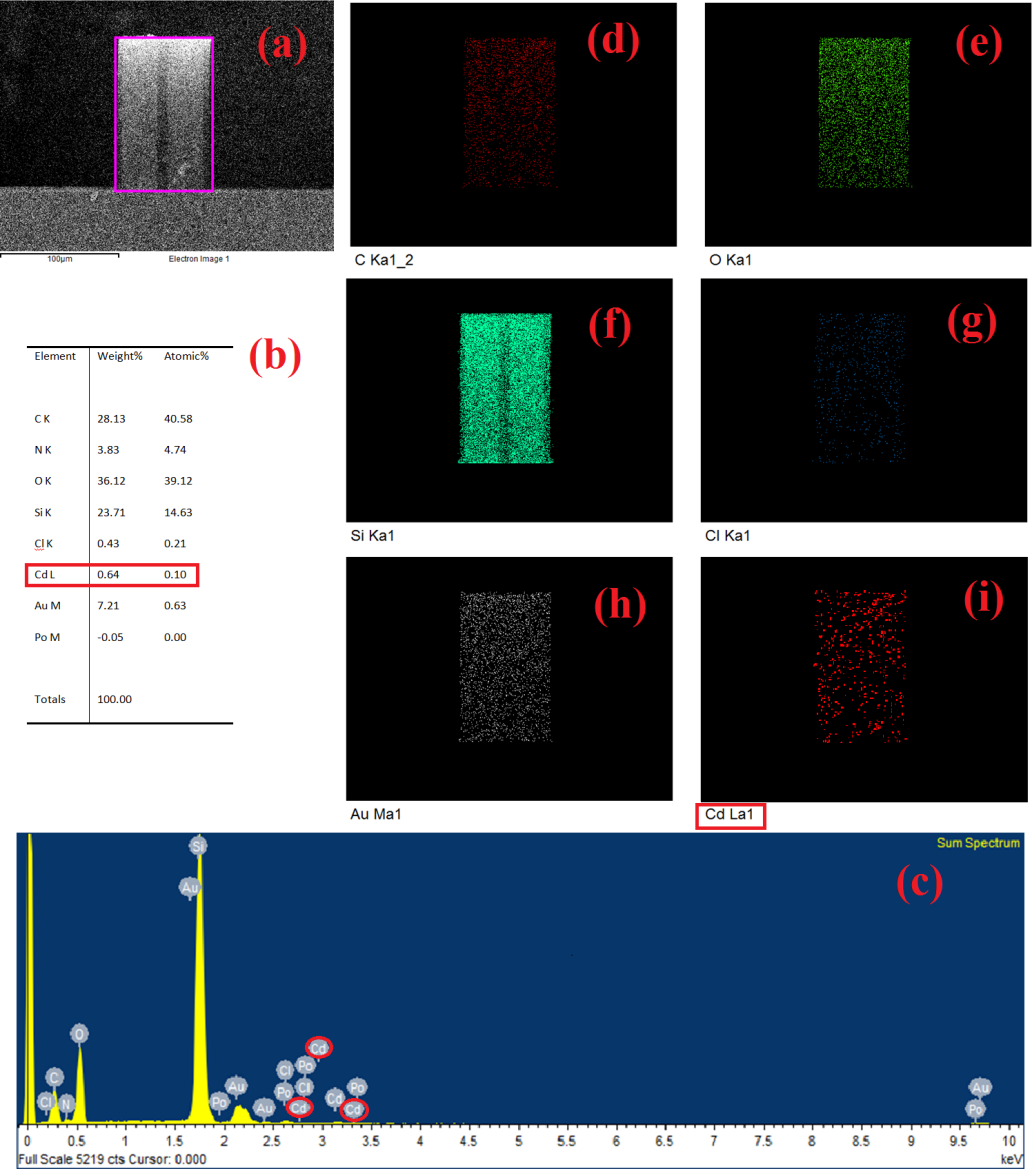
EDX measurement of a SAM of cysteamine (Cys)-cross-linked ᴅʟ-glyceraldehyde (Cys-DL-GC) after exposure to Cd(II). (a) Scan area for analysis, (b) table with detected elements, (c) EDX spectrum, and (d–i) color mappings of detected elements.

The EDX measurement shows that no cadmium ions are detected before exposure to CdCl_2_. The EDX measurement of the microcantilever-based MEMS sensor with SAM of cysteamine cross-linked ᴅʟ-glyceraldehyde (Cys-DL-GC) on top of a Au surface after exposure to CdCl_2_ is shown in Figure 8. The table with the mass percentages (Figure 8b) explicitly shows the presence of Cd(II), and the percentage number of molecules captured in that scan region.

Table 1 presents a comparative analysis of different techniques to selectively capture Cd(II) in the picomolar range. We found that the fabricated piezoresistive sensor needs 20–23 min for selectively capturing Cd(II). At a flow of 30 μL/min, the total injected volume is 0.69 mL for a maximum of 23 min. Therefore, the corresponding mass of AlCl_3_, MnCl_2_, CrCl_3_, CdCl_2_, PbCl_2_, and HgCl_2_ is 0.67 ng, 0.55 ng, 0.74 ng, 0.56 ng, 0.77 ng, and 0.75 ng, respectively (refer to [40,52] for LOD calculation).

**Table 1:** Comparison of different methods for cadmium detection.

reference	Analyte (HMI)	Limit of detection (LOD)	Method used	Detection technique

[4]	Cd(II)	100 pM	microstructured/optical fiber	fluorescence/absorption spectra
[5]	Cd(II)	0.5 nM	ratiometric fluorescence	UV–vis spectroscopy/fluorescence spectra
[32]	Cd(II)	1 μM	carbon paste electrode	XRF/XRD/anodic stripping voltammetry
[33]	Cd(II)	2.15 nM	fluorescent aptamer probe	F-4500/UV-2450 spectrophotometer
[34]	Cd(II)	21 nM	AuNP based probes	colorimetric/FT-IR/DLS
[35]	Cd(II)	10 μM	AuNP based electrode	colorimetric/UV–vis spectroscopy
[38]	Cd(II)	1.33 nM	polymeric-NPs/sol–gel	anodic stripping voltammetry (ASV)/FTIR
[53]	Cd(II)	800 μM	LSPR technique	optical fiber setup
[54]	Cd(II)	2.26 nM	AlGaN/GaN HEMT	high electron mobility transistor (HEMT)
[55]	Cd(II)	65 μM	gold nanoclusters/graphene	fluorescent probe/UV–vis spectroscopy
[56]	Cd(II)	1 μM	gold bioluminescent	fluorescent/microalgae-based
[57]	Cd(II)	1.062 μM	FRET probe-ZnS QD	FTIR/UV–vis/DLS/TEM/(Lab based)
[58]	Cd(II)	5.56 nM	AuNPs-based	colorimetric system/UV–vis spectra/TEM
[59]	Cd(II)	18.5 μM	fluorometric chemosensor	colorimetric/UV–vis/fluorescent spectra
[60]	Cd(II), Pb(II)	2.23 nM	carbon stencil printed electrode	Raman scattering
[61]	Cd(II)	4.95 μM	silver nanoparticles (AgNPs)	UV–vis/FTIR/TEM
[62]	Cd(II), Hg(II)	10–100 pM	electrochemical sensors	PGSTAT potentiostat
[63]	Cd(II), Pb(II)	49.67 pM	cantilever nanobiosensor	atomic force microscope (AFM) setup
[64]	Cd(II)	1 nM	antibody-modified microcantilever	atomic force microscope (AFM) setup
[65]	Pb(II), Cd(II)	1.72–1.58 pM	electrochemical sensor	stripping voltammetry (SWASV)
[66]	Cd(II)	0.3 pM	electrochemical biosensor	AUTOLAB PGSTAT 30
this work	Cd(II)	2.78 pM	piezoresistive sensor	portable setup (real-time analysis)

From the comparison in Table 1, it is clear that the proposed microfluidic platform has the ability to selectively capture Cd(II) at amounts as small as 2.78 pM/mL (LOD) and outperforms other approaches, which require sophisticated measuring instruments. The methods proposed by [4,62–63,65] have outstanding potential for a picomolar range of detection but require costly, sophisticated analytical tools [62–63,65]. The method proposed by [66] has excellent detection in the picomolar range but authors have not studied the sensor response with respect to time, necessary for real-time sensing. Many authors have used colorimetric or fluorescence techniques for selective HMI detection but they are highly sensitive to variation of pH values [67]. In a colorimetric sensor, the concentration variation is shown by different shades of color and it is challenging to exactly identify the shades for the different ranges of concentrations. Both the colorimetric and the fluorescence techniques use NPs and lead to an excessive commercialization of nanoparticles, quickly expanding their harmful effect on life and environment, as discussed earlier.

## Conclusion

The proposed microcantilever-based device was tested in a microfluidic setup for the selective detection of cadmium and was found to achieve sensing in 20–23 min. The 0.56 ng/mL (2.78 pM) limit of detection is possible with a SAM of cysteamine cross-linked ᴅʟ-glyceraldehyde (Cys-DL-GC). The average value of change in piezoresistance of the Au-Cys-DL-GC-coated microcantilever is approximately 130–240 Ω for cadmium ions and 5–30 Ω range for other injected HMIs. The total value of average change in piezoresistance for the concentration of 0.56 ng/mL for Cd(II) is 877.72 Ω. The most significant feature of this approach is the need for a sample volume of one milliliter. It is also evident from EDX spectra that no other HMIs except Cd(II) have been found. This EDX finding shows that the fabricated microcantilever-based piezoresistive sensor does not have cross selectivity. In conclusion, this approach could serve as a portable framework for on-site, ultrasensitive, and selective Cd(II) detection in the picomolar range.
